# Randomized controlled trials of serotonin-norepinephrine reuptake inhibitor in treating major depressive disorder in children and adolescents: a meta-analysis of efficacy and acceptability

**DOI:** 10.1590/1414-431X20164806

**Published:** 2016-05-24

**Authors:** Y. Xu, S.J. Bai, X.H. Lan, B. Qin, T. Huang, P. Xie

**Affiliations:** 1 Department of Neurology, Yongchuan Hospital, Chongqing Medical University, Chongqing, China; 2 Chongqing Key Laboratory of Neurobiology, Chongqing, China; 3 Institute of Neuroscience, Collaborative Innovation Center for Brain Science, Chongqing Medical University, Chongqing, China

**Keywords:** Adolescent, Child, Major depressive disorder, Meta-analysis, Randomized controlled trial, Serotonin and norepinephrine reuptake inhibitor

## Abstract

New generation antidepressant therapies, including serotonin-norepinephrine reuptake inhibitor (SNRIs), were introduced in the late 1980s; however, few comprehensive studies have compared the benefits and risks of various contemporary treatments for major depressive disorder (MDD) in young patients. A comprehensive literature search of PubMed, Cochrane, Embase, Web of Science, and PsycINFO databases was conducted from 1970 to January 2015. Only clinical trials that randomly assigned one SNRI or placebo to patients aged 7 to 18 years who met the diagnostic criteria for major depressive disorder were included. Treatment success, dropout rate, and suicidal ideation/attempt outcomes were measured. Primary efficacy was determined by pooling the risk ratios (RRs) of treatment response and remission. Acceptability was determined by pooling the RRs of dropouts for all reasons and for adverse effects as well as suicide-risk outcomes. Five trials with a total of 973 patients were included. SNRIs were not significantly more effective than placebo for treatment response but were for remission. The comparison of patients taking SNRIs that dropped out for all reasons and those taking placebo did not reach statistical significance. Significantly more patients taking SNRIs dropped out for adverse effects than those taking placebo. No significant difference was found in suicide-related risk outcomes. SNRI therapy does not display a superior efficacy and is not better tolerated compared to placebo in these young patients. However, duloxetine has a potential beneficial effect for depression in young populations, showing a need for further research.

## Introduction

The treatment of depression in children and youths is a major public health problem. Unfortunately, few well-controlled, large-scale, randomized clinical trials have been conducted in this population. Its point prevalence is estimated to be about 1–2% for children (6–12 years), 2–8% for adolescents (13–18 years) and 6–9% in young adults (19–25 years) experiencing at least one episode of major depression before adulthood (1–3). As with adults, the course of major depression in children and adolescents is often characterized by protracted episodes, frequent recurrence, comorbidity with psychiatric disorders, and impairment in academic domains and family and peer relationships (4,5). Childhood and adolescent major depressive disorder (MDD), or unipolar major depression, is responsible for extensive morbidity and mortality, with depression-related suicide ranking as the third leading cause of adolescent death (6). Though suicide completion rates remain low in pediatric depression patients, suicidal ideation and attempts are common (7).

For depression in children and adolescents, non-pharmacological interventions, such as cognitive-behavioral therapy and other psychotherapies, have been shown to be effective in improving the symptoms, at least in mild or moderate depressive disorders (8,9). For severe or resistant depression, pharmacological treatment may be needed. Nevertheless, the prescription prevalence in children and adolescents has been increasing in the last few years (10–12). Due to the poor efficacy of tricyclic drugs and their association with significant adverse effects (13), selective serotonin reuptake inhibitors (SSRIs) are used more frequently, even though their risk-benefit profile has been the basis of debate among both the scientific community and lay people since 2000 (14).

In addition to SSRIs, several other classes of antidepressants are now being used, including selective norepinephrine reuptake inhibitors (SNRIs), norepinephrine reuptake inhibitors (NRIs), norepinephrine dopamine reuptake inhibitors (NDRIs), norepinephrine dopamine disinhibitors (NDDIs), and tetracyclic antidepressants (TeCAs) (15). New therapies with improved efficacy and similar or better tolerability as compared with SSRIs would be valuable additions to depression therapy (16). Evidence from several sources suggests that (SNRIs) - such as venlafaxine, duloxetine, desvenlafaxine, and milnacipran - may be more effective treatments for depression than therapies that act on a single neurotransmitter (17,18). Therefore, the purpose of this study was to evaluate the efficacy and safety of SNRIs in children (7–11 years) and adolescents (12–18 years) with MDD. This is the first meta-analysis analyzing the efficacy of SNRIs in MDD children and adolescents.

## Material and Methods

### Search strategy

A comprehensive search of PubMed, Cochrane, Embase, Web of Science, and PsychINFO databases from 1970 through January 2015 was conducted with the following search terms: (SNRI* or venlafaxine or duloxetine or desvenlafaxine or milnacipran or “selective norepinephrine reuptake inhibitor”) and (depression* or depressive or depressed) and (adolesc* or child* or boy* or girl* or juvenil* or minor or paediatri* or pediatri* or pubescen* or school* or student* or teen* or young or youth*). No language restrictions were applied. Additional trials were obtained by scanning reference lists of all identified records.

### Definitions and inclusion criteria

Two authors (Y. Xu and B. Qin) independently screened the titles and abstracts of each citation and selected studies based on the following inclusion criteria: *i*) clinical trials enrolling children and adolescents with a current unipolar depressive disorder; *ii*) clinical trials with random assignment comparing an SNRI with a placebo, and *iii*) clinical trials reporting depressive symptom outcome(s). We excluded trials with duplicate secondary analyses or bipolar depression patients. Any disagreements were resolved via discussion and arbitration by a third reviewer if necessary.

### Bias risk in individual studies

Bias risk was determined by: *i*) randomization quality; *ii*) allocation concealment; *iii*) blinding of outcome assessment; *iv*) incomplete reporting of outcome data, and *v*) similarity in baseline clinical characteristics. Studies with three or more bias risks were excluded from the meta-analysis.

### Outcome measurement

For both efficacy and acceptability analyses, acute treatment was defined as an 8-week treatment period. If 8-week data were not available, data ranging between 6 and 12 weeks were used (with preference to the time point given in the original study as the study endpoint). The most common measure of antidepressant efficacy used in clinical trials has been the response rate. Response is defined as a 50% reduction from baseline in depression scale scores (19). A more stringent measure of antidepressant efficacy is remission, which is a clinical state characterized by minimal residual symptoms (20). Clinical remission is typically defined as a score that lies clearly within the normal range on the various scales used to measure depression. Accepted scores for remission are ≤7–8 on the HAMD-17 scale or ≤10–12 on the MADRS scale. Fava et al. (21) stated that patients treated to full remission were less likely to relapse than were non-remitted patients. Similarly, Miller et al. (22) found that remitters had more normal psychological and vocational functioning. When a study reported multiple depression rating scales, the Childhood Rating Depression Scale-Revised (CRDS-R) was used.

Primary acceptability (tolerability) was defined as the proportion of patients who prematurely terminated the study for any reason. For secondary acceptability (safety) measures, the proportion of subjects who prematurely terminated the study for side effects. The suicide-related outcome was calculated as the proportion of patients who experienced one or more events of definite suicidal ideation or behavior. If possible, we also extracted over all adverse outcomes (number with any adverse outcome reported).

### Statistical analysis

We performed a pairwise meta-analysis using Review Manager (version 5.2, Cochrane, Denmark) and Stata (v11.0, StataCorp, USA). Because of the varying clinical conditions and settings represented in the studies, we expected that data sets with one efficacy would be heterogeneous. When possible, we used the intention-to-treat population for analyses. The heterogeneity of treatment effects across studies was assessed by I^2^ and the Cochrane Q test (23). Publication bias was examined with the funnel plot method, Begg's adjusted rank correlation test, and Egger's regression asymmetry test (24,25). We conducted one subgroup analyses to examine whether effect estimates would be influenced by the type of SNRI investigated in the individual trials. We also performed sensitivity analyses after the omission of data from low dose studies. For heterogeneous data, we used a random effects model; otherwise, a fixed effect model was used.

## Results

### Study selection and characteristics

**Figure 1:**
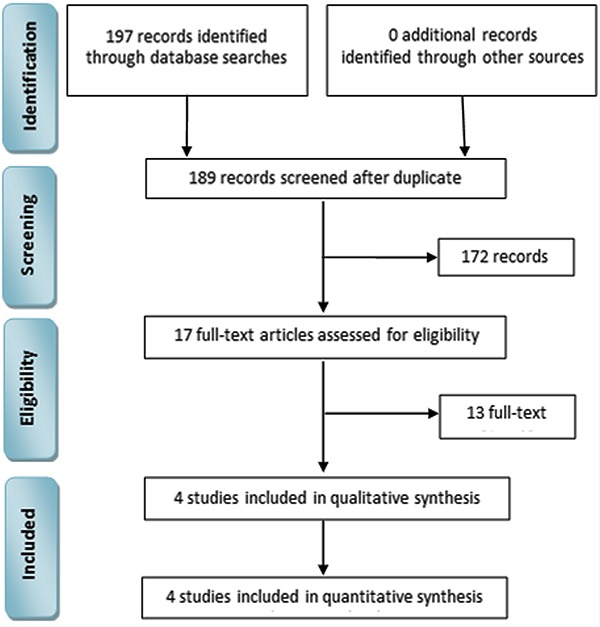
Identification process for study inclusion in the meta-analysis.

The identification of studies for inclusion in the meta-analysis is reported in Figure 1. We initially retrieved 197 potentially relevant studies. Of these, 180 articles were excluded because their titles and abstracts did not meet the inclusion criteria. A total of 13 studies were excluded after two reviewers independently read the full texts (Y.X; B.Q.) because some were repeated publications or system analyses and others had no valid data. Thus, four studies (five trials) consisting of 973 patients were considered for inclusion in the meta-analysis (26–29). Patients in the five randomized clinical trials (RCTs) were from the United States, Finland, France, Germany, Slovakia, Estonia, Russia, Ukraine, and South Africa. Table 1 summarizes the clinical and methodological characteristics as well as the main outcomes of each trial. The trials were published between 1997 and 2014. The overall male/female ratio was 0.49. The age range of participants was 7–17 years. All included studies recruited patients who were rated as having MDD. The median duration of studies was 8.6 weeks (range: 6–10 weeks), which was a sufficient duration for antidepressant effects to occur. The studies involved two different SSRIs: duloxetine (3 trials) and venlafaxine (2 trials). The duloxetine doses ranged from 30 to 120 mg/day. A flexible venlafaxine ER dose based on body weight was used in one study, while the other used a different fixed venlafaxine dose schedule for children and adolescents. In addition, SNRIs were represented only by venlafaxine and duloxetine. We did not locate any studies of the SNRI milnacipran.

**Table:**
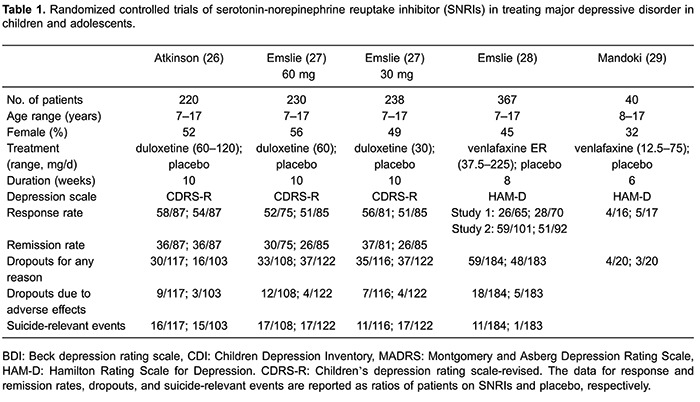

### Bias risk assessment

All five included RCTs were randomized, concealed allocation, and reported similar baseline characteristics. Although all the RCTs had patients who withdrew, all reported incomplete data. Four RCTs unequivocally reported the outcomes of their blinding assessment. As the five RCTs displayed minimal or no bias risk, the data from all studies were included in this meta-analysis.

### Response rates

**Figure 2:**
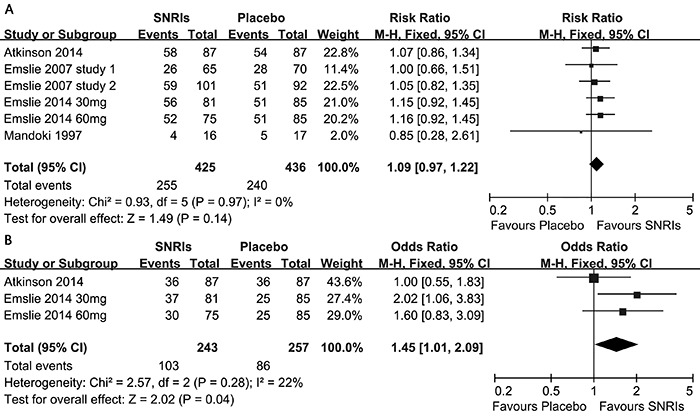
Primary outcome and secondary outcome: serotonin-norepinephrine reuptake inhibitor (SNRIs) *vs* placebo paradigms. *A*, comparison of SNRIs *vs* placebo for the primary outcome (response at end of treatment). *B*, comparison of SNRIs *vs* placebo for the secondary outcome (remission at end of treatment). See Table 1 for details of references.

Response rates at the treatment endpoint were available for all five RCTs (Figure 2A). In these trials, 255 of 425 SNRIs-treated subjects (60%) and 240 of 436 placebo-treated subjects (55%) responded. The pooled odds ratio (OR) was 1.09 (95%CI=0.97–1.22, z=1.49, P=0.14), indicating a comparative efficiency between SNRIs and placebo. There was no significant heterogeneity in effect size (P=0.97, I^2^=0%). Furthermore, significant asymmetry was non-existent in the inverted funnel plots of these trials. Considering that the number of selected trails may have not provided enough power to show clear asymmetry, Egger's test was performed and showed that the primary outcome (P=0.24) was not influenced by publication bias.

### Remission rates

Remission rates at the treatment endpoint were available for three RCTs (Figure 2B). In these trials, the remission rates varied between 41 and 46% in the duloxetine groups and between 30 and 41% in the placebo groups. A total of 103 of 243 SNRI-treated subjects (42%) and 86 of 275 placebo-treated subjects (31%) remitted. The pooled OR was 1.45 (95%CI=1.01–2.09, z=2.02, P=0.04), indicating a comparative efficacy between SNRIs and the placebo. There was significant heterogeneity in effect size (P=0.28, I^2^=22%).

### Acceptability outcomes

**Figure 3:**
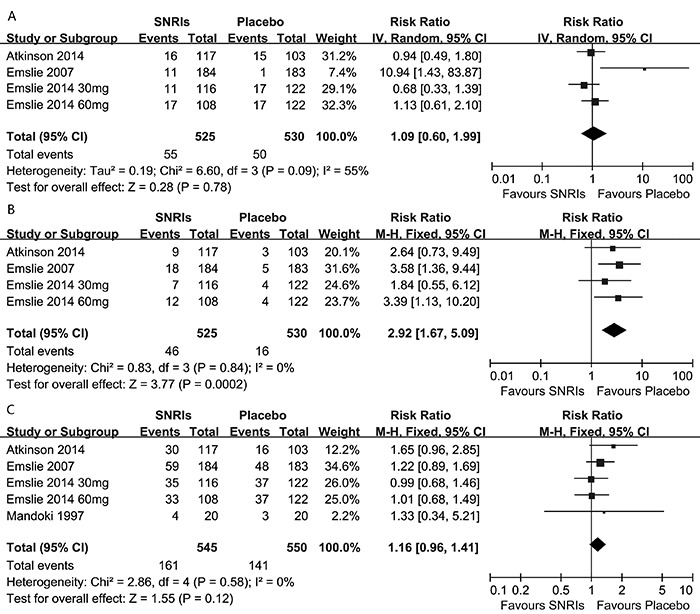
Acceptability outcomes: serotonin-norepinephrine reuptake inhibitor (SNRIs) *vs* placebo paradigms. *A,* comparison of SNRIs *vs* placebo for suicide-related outcome. *B,* comparison of SNRIs *vs* placebo for the outcome (patients discontinued treatment due to adverse effects). *C,* comparison of SNRIs *vs* placebo for the outcome (patients discontinued treatment due to reasons unrelated to adverse effects). See Table 1 for details of references.

The data on the primary acceptability outcomes are shown in Figure 3. More patients on SNRIs therapy dropped out for specific reasons than those on placebo (29.5 *vs* 25.6%), although this comparison did not reach statistical significance (RR=1.16, 95%CI=0.96–1.41, P=0.12; Figure 3C). Significantly more patients on SNRI therapy dropped out for adverse effects than those on placebo (8.8 *vs* 3.0%; RR=2.92, 95%CI=1.67–5.09, P=0.0002; Figure 3B).

### Suicide-related outcomes

No significant difference was found in suicide-related risk outcomes for those receiving SNRIs compared with those receiving placebo (five trials; RR=1.09; 95%CI=0.60–1.99; P=0.78; Figure 3A).

### Subgroup analysis

A subgroup analysis was conducted in order to compare the efficacy and acceptability of placebo against duloxetine or venlafaxine. With regard to response, three studies compared duloxetine to placebo, and three studies compared venlafaxine to placebo. No significant difference was found in either comparison. The OR for duloxetine to placebo was 1.13 (95%CI=0.99–1.28), and the OR for venlafaxine to placebo was 1.03 (95%CI=0.83–1.27). With respect to dropouts for adverse effects, no significant difference was found in either the duloxetine versus placebo comparison or the venlafaxine versus placebo comparison. The OR of the former was 2.59 (95%CI=1.30–5.13), and the OR of the latter was 3.58 (95%CI=1.36–9.44). With respect to suicide-related outcomes, no significant difference was found in the duloxetine comparison. The OR of duloxetine to placebo was 0.92 (95%CI=0.63–1.34). However, there was evidence of an increased risk of suicide-related outcomes for those taking venlafaxine compared with placebo, although there were few suicide-related events and the resulting CI was very wide. The OR for venlafaxine to placebo was 10.94 (95%CI=1.43–83.87).

### Overall adverse outcomes

For the venlafaxine trials, there were no data on the number of overall adverse events experienced by young people in these trials. Data on individual adverse events highlighted that abdominal pain and dizziness were reported more often with treatment than with placebo. For the duloxetine trials, the most frequently reported TEAEs (≥10%) during the study were: nausea, headache, and nasopharyngitis.

## Discussion

To our knowledge, this meta-analysis is the first pairwise comparison of efficacy and acceptability between SNRIs and placebo in children and adolescents. A total of four studies (five RCTs), which consisted of 970 patients, on the effects of SNRI treatment in children and adolescents with MDD were finally identified in this systematic review and meta-analysis.

Increasing evidence suggests that, in some depressed patients, SNRIs may provide the benefits of treating a broader range of target symptoms than single-acting agents, such as SSRIs. A previous review (30) provides evidence that duloxetine 60 mg QD is effective for the treatment of adult patients with MDD in both the short-term and long-term phases of treatment and indicates that venlafaxine XR is an efficient and safe therapeutic option for patients with MDD with the additional effect of reducing associated painful physical symptoms. However, we cannot apply findings from adult inpatients to a younger population. Moreover, methodological deficiencies in the previous review, including small sample sizes and pilot studies, limit the statistical inference and generalizability of the findings. In addition, the selection bias of unpublished data was another important deficiency in the previous review, as the authors reported an equivalence between SNRIs and placebos when both published and unpublished data were considered. In this study, we grouped two SSRIs: venlafaxine and duloxetine. Subgroup analysis showed that venlafaxine not only had no effect, but also raised suicide risk. Though there were limited cases in the venlafaxine study, some other reports have also demonstrated that venlafaxine increases the risk of suicide (14,31,32). There was evidence of increased risk of suicide-related outcomes for those receiving venlafaxine compared with placebo, although there were few suicide-related events and the resulting CI was very wide (14). But, subgroup analysis of our data showed that duloxetine (only two RCTs) has a potential effect for depression in young populations. However, more research on duloxetine for MDD in adolescents is needed.

More patients dropped out for reasons unrelated to adverse effects from SNRIs than from placebo treatment, although this comparison did not reach statistical significance; however, significantly more patients on SNRI therapy dropped out for adverse effects than those on placebo. In addition, patients undergoing SNRI therapy and placebo treatment had similar suicide-related rates.

Therefore, the overall findings of these studies do not suggest that venlafaxine ER should be a first-line treatment for depression in young populations. However, duloxetine has a potential effect for depression in young populations. In addition, the subgroup analysis showed that the effect sizes in studies with small sample sizes seem to be higher than those with large sample sizes, and this subgroup analysis decreased the heterogeneity within the groups. Statisticians have previously demonstrated that a small sample size may increase the variability of results by producing a larger standard deviation, thereby affecting study reliability by overstating medication effects (33). Methodologists and authors of systematic reviews have also stated that many effects have been mixed due to failures in study planning and inadequate sample sizes (34).

### Limitations

Despite the increasing number of randomized trials assessing antidepressants for MDD in recent years, the total number of studies and patients randomly assigned for children and adolescents remains low. Thus, only five trials met our inclusion criteria, and some studies included in this review showed small sample sizes and limited statistical power. However, combining these studies resulted in a relatively high power to estimate treatment effects. The variation in sample characteristics and trial features across the studies (e.g., age ranges from children to young adults, different doses of one antidepressant) can be viewed as a strength with respect to study generalizability, but these factors can also hide potential bias. Thus, future studies should consist of larger sample sizes and multi-center designs in order to isolate and examine factors such as age grouping and gender.

These findings indicate that venlafaxine ER does not display a superior efficacy and is not better tolerated compared to placebo in young patients. Moreover, there was evidence of an increased risk of suicide-related outcomes for those receiving venlafaxine compared with those receiving placebo; therefore, venlafaxine might not be a wise recommendation for children and adolescents with MDD. On the other hand, duloxetine might have a potential beneficial effect for depression in young MDD populations, although there is a need for further research. In order to more robustly examine SNRI efficacy in these young patients, future studies should be planed with larger sample sizes and multi-center designs, so factors such as age grouping and gender could be isolated and examined.
